# The Association Between Frequency of Social Media Use, Wellbeing, and Depressive Symptoms: Disentangling Genetic and Environmental Factors

**DOI:** 10.1007/s10519-025-10224-2

**Published:** 2025-06-21

**Authors:** Selim Sametoğlu, Dirk H. M. Pelt, Meike Bartels

**Affiliations:** 1 Max Planck Institute for Psycholinguistics, Nijmegen, Netherlands; 2 Vrije Universiteit Amsterdam, Amsterdam, Netherlands; 3 Amsterdam Public Health Research Institute, Amsterdam, Netherlands

**Keywords:** Classical twin design, Genetically informed designs, Social media use, Wellbeing

## Abstract

Meta-analyses report small to moderate effect sizes or inconsistent associations (usually around *r* = -0.10) between wellbeing (WB) and social media use (SMU) and between anxious-depressive symptoms (ADS) and SMU (also around *r* = 0.10). This study employs the classical twin design, utilizing data from 6492 individuals from the Netherlands Twin Register, including 3369 MZ twins (893 complete twin pairs, 1583 incomplete twin pairs) and 3123 DZ twins (445 complete, 2233 incomplete) to provide insights into the sources of overlap between WB/ADS and SMU. Both hedonic and eudaimonic WB scales were used. SMU was measured by (1) the time spent on different social media platforms (SMU_t_), (2) the frequency of posting on social media (SMU_f_), and (3) the number of social media accounts individuals have (SMU_n_). Our results confirmed the low phenotypic correlations between WB and SMU (between *r* = -0.09 and 0.04) as well as between ADS and SMU (between *r* = 0.07 and 0.10). For SMU, heritability estimates between 32 and 72% were obtained. The small but significant phenotypic correlations between WB/ADS and the SMU phenotypes were mainly determined by genetic factors (in the range of 80-90%). For WB and SMU, genetic correlations were between -0.10 and -0.0, and for ADS and SMU genetic correlations were between 0.10 and 0.23. Genetic correlations implied limited but statistically significant sets of genes that affect WB/ADS and SMU levels. Overall, the results indicate that there is evidence that the small associations between WB/ADS and SMU are partly driven by overlapping genetic influences. We encourage researchers and experts to consider more personalized approaches when considering the association between WB and SMU, as well as understanding the reasons for individuals’ observed SMU levels.

**Supplementary Information:**

The online version contains supplementary material available at 10.1007/s10519-025-10224-2.

## Introduction

As of April 2023, there are 4.80 billion social media users, equivalent to 59.9% of the global population. Individuals daily spend on average around 2 and a half hours on social media platforms (Datareportal 2023). Over the last decade, the number of people using social media platforms has been consistently growing. Public and governmental organizations are paying more attention to social media use, and its potential positive and negative effects on individuals, such as their wellbeing and mental health (Pereira et al. 2021; Izutsu et al. 2015).Wellbeing can be broadly categorized as subjective (or hedonic) and psychological (or eudaimonic) wellbeing (Deci and Ryan 2008; Ryff 1989). Hedonic wellbeing consists of cognitive and affective evaluations of one’s life, whereas eudaimonic wellbeing refers to positive functioning which entails multiple domains such as having positive relations, autonomy, environmental mastery, positive growth, purpose in life, and self-acceptance (Ryff 1989). Higher levels of wellbeing are associated with a wide range of positive outcomes such as having better social relations, and finances, as well as higher school achievement, altruistic behaviour, and workplace functioning (Chapman and Guven 2016; James et al. 2019; Maccagnan et al. 2019; Okabe-Miyamoto & Lyubomirsky, in press; Oswald et al. 2015; Steptoe 2019; Walsh et al. 2018). At the nation level, higher wellbeing is associated with higher GDP, lower healthcare costs and sickness benefits (Hagerty and Veenhoven 2003; Santini et al. 2021a; Santini, Nielsen, Santini et al. 2021a, b).Similarly, depressive-anxious symptoms indicate a burden to society and individuals, given their association with increased healthcare costs in public health systems (Vasiliadis et al. 2013), poverty (Ridley et al. 2020), suicidal ideation (Casey et al. 2008; Spijker et al. 2010), and loneliness (Lee et al. 2021) among many negative outcomes.

### Association Between Wellbeing/anxious-Depressive Symptoms and Social Media Use

In recent years, there has been a strong increase in the number of studies on the association between social media use with wellbeing (WB) and anxious-depressive symptoms (ADS) (Valkenburg 2022). For instance, multiple meta-review studies, combining results of the meta-analyses, indicated either small to moderate effect sizes or inconsistent results (Appel et al. 2020; Valkenburg 2022; Valkenburg et al. 2022a, 2022b). The most recent meta-review included 27 review studies on SMU and WB/ADS published between 2019 and 2021 (Valkenburg 2022). This study reported that six out of seven meta-analyses found effect sizes that ranged from very small to moderate (absolute correlations, *r* = 0.05–0.17), while one meta-analysis reported mainly null results (Cunningham et al. 2021).

Overall, the lack of consistent results has been partly attributed to overreliance on cross-sectional research (e.g., Parry et al. 2022; Valkenburg et al. 2022a). As a potential solution to this, multiple studies used longitudinal data sets. For example, a study found small negative associations for WB with retrospectively reported and diary-logged digital screen engagement as a measure of SMU (*r* = − 0.08, and *r =* −0.02, respectively) (Orben and Przybylski 2019). Similarly, another longitudinal study investigated ADS and SMU of a group of individuals from 10 to 16 years with 2-year lags. The results indicated null within-person level associations and a few small (between *r* = 0.04–0.07) associations at the between-person level (Steinsbekk et al. 2023). Other longitudinal studies applied a more person-centric approach by investigating whether the associations differed for each individual. Their results showed that the association between WB/ADS and SMU differs in both direction and magnitude among individuals over time (Beyens et al. 2020; Pouwels et al. 2021).

It has also been considered whether different ways of assessing social media use could lead to different results (Hogue and Mills 2019; Verduyn et al. 2017, 2022). Social media use can be categorised in active social media use (e.g., posting frequency, sending messages) or passive usage measures (e.g., scrolling), in which passive SMU is considered to have a negative influence on wellbeing, whereas active SMU’s influence is considered to be less conclusive. For instance, a study using a population sample of Icelandic adolescents (*N* = 10,563) reported increased passive social media use to be associated with increased anxiety and depressive symptoms, effects that persisted after controlling for time spent on social media. Another study, in a sample of adults between 18 and 49 age (*N* = 702), reported an association between increased passive SMU and negative wellbeing, but reported the opposite results for active SMU (Escobar-Viera et al. 2018). Further, although active and passive SMU distinctions in the assessment of SMU exist, there is no consensus on the usefulness of active versus passive social media usage measures in the field (Valkenburget al. 2022c; Verduyn et al. 2017, 2022).

### Genetically Informed Designs

Overall, evidence indicates that small associations between WB/ADS and SMU are present. Furthermore, most studies assume the direction of the association to be from increased SMU to decreased WB/increased ADS, yet substantial evidence to claim the direction of causation is missing given the cross-sectional nature of most studies in this field. Besides the size and the direction of the associations between WB/ADS and SMU variables, it is important to study the underlying sources of the associations between WB/ADS and SMU. The extent to which the phenotypic association between two variables is partly due to overlapping genetic factors that influence both WB/ADS and SMU can be investigated by leveraging genetically informed designs.

As an example of such genetically informative designs, the classical twin design can be used. Classical twin designs are based on data from monozygotic (MZ) and dizygotic (DZ) twins, which allow for disentangling the effects of genetic and environmental influences on the observed covariance between WB/ADS and SMU (e.g., De Vries et al. 2021). In addition to estimating the underlying sources of covariance, multivariate models provide the opportunity to calculate the genetic and environmental correlations that indicate the overlap in genetic and environmental factors between phenotypes.

For the relation between WB/ADS and SMU to be partly driven by genetics, it is a prerequisite that each of these traits is heritable. Evidence based on genetically informed designs (e.g., twin studies, molecular genetic studies) indicates individual differences in WB, ADS, and SMU to be partly accounted for by genetic factors. For instance, heritability estimates based on twin studies for WB are 40–50% (for a review and meta-analysis see, Bartels 2015; Nes and Røysamb 2017; Van De Weijer et al. 2022). Similarly, for ADS, twin-based heritability estimates ranging from 49 to 60% are found throughout childhood into adulthood in one study (Baselmans et al. 2018), supported by heritability estimates of 37% found in a meta-analysis (Sullivan et al. 2000) and a recent review study which has mentioned heritability estimates ranging between 30 and 50% (Kendall et al. 2021).

Although few studies have examined the heritability of social media use specifically, related research on broader constructs such as general internet and media use, problematic internet use (e.g., cyberbullying), and internet addiction (i.e., excessive or maladaptive internet use) offers valuable insights. Heritability estimates for problematic internet use have been reported to range from 21 to 66% (Ayorech et al. 2023; Deryakulu & Ursavas, 2014; Hahn et al. 2017; Li et al. 2014). Similarly, studies investigating general internet and social media use report heritability estimates between 24% and 67% (Ayorech et al. 2017, 2023; Long et al. 2015; York 2017). Among these, Ayorech et al. (2017) is particularly noteworthy, as it provides univariate heritability estimates specifically for social media (Facebook) use. In this study, social media use was operationalized as a composite of self-reported metrics: the duration of having a Facebook account, frequency of checking Facebook updates, and number of Facebook friends. This composite measure yielded a heritability estimate of 24%, with a confidence interval ranging from 17 to 32%.

In terms of bivariate analyses examining the relationship between social media use and wellbeing, the study by Ayorech et al. (2023) is particularly relevant. This work investigated the genetic and environmental overlap between general media use and prosocial behavior, a construct conceptualized as a positive indicator of wellbeing, in a sample of emerging adult twins. General media use in their study encompassed a wide spectrum of behaviors, including text messaging, emailing, video gaming, Facebook friendships, television watching, and both positive and negative attitudes toward media. Although not focused solely on social media use, their findings revealed a phenotypic correlation of *r* = 0.19 between general media use and prosocial behavior. 88% of this association was attributed to additive genetic factors, highlighting a substantial genetic contribution to the overlap between media engagement and wellbeing-related traits.

### The Present Study

In the present study, we apply a multivariate twin design to disentangle additive genetic and environmental influences—both shared and non-shared—on the observed associations between wellbeing (WB), depressive symptoms (ADS), and social media use (SMU). Additionally, we compute genetic and environmental correlations to quantify the degree of overlap in the underlying factors contributing to these phenotypes.

In doing so, we aim to build upon the limited but growing body of research investigating the genetic and environmental underpinnings of the link between positive wellbeing and social media use (e.g., Ayorech et al. 2023). Our study extends this work in several key ways. First, we focus explicitly on social media use by aggregating multiple usage characteristics across popular platforms (e.g., Facebook, Snapchat), without conflating these behaviors with broader online media activities such as gaming or television viewing. To capture social media use from multiple angles, we include measures of time spent on platforms (SMU_t_), posting frequency (SMU_f_), and the number of accounts maintained (SMU_n_).

With respect to wellbeing, we adopt a comprehensive approach by assessing both hedonic and eudaimonic components, alongside a distinct measure of illbeing (ADS), following recommendations by Valkenburg (2022). Moreover, unlike previous research that has largely focused on adolescents or young adults (e.g., Ayorech et al. 2017, 2023; Deryakulu & Ursavas, 2014; Li et al. 2014), our study draws on a large population sample spanning a wider age range. Given prior evidence that the associations between social media use and wellbeing or illbeing may vary by sex and age—with younger individuals and females potentially more susceptible to negative effects (e.g., Orben et al. 2022) we include both age and sex as covariates in our analyses, leveraging the full diversity of our sample.

## Method

The description of the dataset and the planned analyses were preregistered before analysing the dataset. The analysis plan and the (arguments for) deviations from this plan can be found on the Open Science Foundation (OSF) website (https://osf.io/jr4xc/).

### Sample and Participants

The data used in this study were obtained from the Netherlands Twin Register (NTR; Ligthart et al. 2019) after meeting the requirements posited by the NTR Data Access Committee and obtaining their permission. In total, the sample included 6492 individuals, including 3369 MZ twins (893 complete twin pairs, 1583 incomplete twin pairs) and 3123 DZ twins (445 complete, 2233 incomplete). The mean age in the sample was 35.10 (*SD* = 14.93, range = 16–89). The sample consisted of 71% females (*n* = 4618).

### Measures

**Hedonic wellbeing -** Hedonic wellbeing was assessed with the satisfaction with life scale (SWL, Diener et al. 1985), the subjective happiness scale (SHS, Lyubomirsky and Lepper 1999), and a single-item measure of quality of life (QoL, Cantril 1965). The SWL scale consisted of five items and the SHS consisted of four items (the fourth item was reverse-coded). Both measures were responded to on a 7-point Likert scale with answer options ranging from strongly disagree to strongly agree. An example item for satisfaction with life scale is: “I am satisfied with my life.” An example item for the subjective happiness scale is “Compared with most of my peers, I am less happy than they are.” The items were summed to obtain an overall SWL and SHS score.

The exact item used for QoL consisted of the following two sentences: “Where on the scale would you put your life in general? A score of 10 means the best life you can imagine, and zero means the worst life you can imagine”. The participants provided their responses by using a ten-step ladder. In the present sample, the reliability of the SWL and SH scales were 0.87 and 0.89, respectively. Single-item measures for wellbeing (such as Cantril Ladder in this case) are usually found reliable (Lucas and Brent Donnellan 2012).

**Eudaimonic wellbeing** - Eudaimonic wellbeing was assessed by calculating a sum of responses on the Flourishing Scale (FL; Diener et al. 2010). The scale consists of eight items that could be responded to on a 7-point Likert scale with answer options ranging from strongly disagree to strongly agree. An example item was: “I am engaged and interested in my daily activities.” In the present sample, the reliability of the FL scale was 0.90.

**Anxious-depressive symptoms -** The anxious-depressive symptoms score (ADS) was assessed by calculating a sum score based on the 18 items from the Adults Self-Report Anxious-Depressed Syndrome Scale (Achenbach and Rescorla 2003). The responses were given on a three-point Likert scale: (1) “Not at all”, (2) “somewhat or sometimes”, and (3) “very much or so often”. An example item was: “I worry about my future”. In the present sample, the reliability of the ADS scale was 0.92.

**Time spent on different social media platforms (SMU**_**t**_**) -** was assessed by calculating the sum value of the responses obtained separately for five different social media platforms - Facebook, Instagram, Snapchat, Twitter, and LinkedIn. The exact item used in this study was: “How much time do you spend on ‘[PLATFORM NAME]’?”. The response options were on a 6-point Likert scale: 1-“never”, 2-“less than 30 minutes”, 3-“30 to 60 minutes”, 4-“1 to 2 hours”, 5- “2 to 3 hours”, 6- “more than 3 hours”.

**The frequency of posting on social media (SMU**_**f**_**) -** was measured by a single item: “How often do you post on social media?” The response options were on a 6-point Likert scale: (1) “Never”, (2) “once every couple of months”, (3) “once every couple of weeks”, (4) “weekly”, (5) “daily”, (6) “multiple times a day”. We merged the categories (5) “daily” and (6) “multiple times a day” with each other as the responses provided for these categories (149 and 39) were considerably lower than the other categories (which were 1330, 1704, 1081, 396 from “1” to “4”, respectively). The new category was labelled as (5) “daily”.

**The number of social media accounts (SMU**_**n**_**)** was measured by a single item. The exact item used in this study was: “On how many social media channels do you have an account (e.g., Facebook, Instagram, Twitter, LinkedIn, not Whatsapp)?”. The participants were allowed to respond through one of the eight following options: “0”, “1”, “2”, “3”, “4”, “5”, “6”, “7”, “8 or more”. We merged the last 2 answer categories (“7”, and “8 or more”) with the response category “6” given the number of individuals who selected these last two answer categories was noticeably more limited (25 and 87, respectively) than the responsesto other categories (which were 361, 810, 1058, 748, 356, 119 from “0” to “6”, respectively).

## Analyses

Twin models leverage the differences in the degree of relatedness between monozygotic twin pairs (sharing 100% of the segregating genes) and dizygotic twin pairs (sharing on average 50%) to decompose the variance of a phenotype and the covariance between phenotypes in genetic and environmental variance/covariances. Additive genetic variance (A) indicates the proportion of variance that can be explained by the effect of independent alleles on the phenotype. Non-additive genetic variance (D) indicates the proportion of variance explained by interactions between alleles at the same locus (dominance) or between alleles from different loci (epistasis). Environmental variance consists of environmental variance shared by members of the same family (C), and a unique environment component (E) including individual-specific environmental influences and measurement error.

In the classical twin model, models are not identified when both the C and D variance components are included, therefore a decision has to be made based on observed data and/or results from previous studies. In the observed data, if the cross-twin-cross-trait correlations for MZ twins are more than 2 times the correlations for DZ twins (i.e., *r*MZ > 2*rDZ), this is an indication of dominant genetic effects, whereas if the opposite is true (i.e., *r*MZ < 2*rDZ), shared environment effects are more plausible.

We first estimated phenotypic correlations, twin correlations, and cross-twin cross-trait with a saturated model using the OpenMx package (Boker et al. 2011) in R (R Core Team 2000). We treated missing values in our data using the Full Information Maximum Likelihood (FIML) method. In the saturated model, we included all of the eight phenotypic variables, namely the four wellbeing scores, one anxious-depressive symptom score, and the three social media use scores. Age was included as a covariate on means given the presence of age differences in social media use (Datareportal 2023). The age variable was divided by 100 to facilitate model estimation, which means that the regression coefficients are expressed in centuries. Sex is known to be a potential moderator for explaining the associations between WB/ADS and SMU (Kelly et al. 2018; Nesi and Prinstein 2015). However, in the present study, the sample size was too small for testing sex differences; therefore, sex was only included as a covariate on means, similar to age.

Overall, our analyses were based on two zygosity groups (MZ and DZ twins). First, for each phenotype, we tested in a univariate model whether the assumption of equal means and variances for the oldest and youngest twins and for the MZ and the DZ twins held. All versions of the saturated models, with a unique set of equality constraints on various parameters, were compared to the initial saturated model without any of the constraints. These comparisons were made using the chi-square (*χ*2) difference test. If the model fit changes significantly when constraints are introduced (as indicated by a significant χ² difference test result at *p* < 0.01), this suggests that the tested assumption in the constrained model was not successfully met. In addition, we utilised the AIC values between the models for comparison for both nested and non-nested models to assess model fit (smaller values indicated better fit). After checking the classical twin model assumptions, we estimated cross-twin cross-trait correlations in an 8-variate saturated model, with sex and age as the covariates on the means.

## Cholesky Model

Based on the cross-twin cross-trait correlations, we applied a Cholesky decomposition to decompose the phenotypic covariance matrix into genetic and environmental (co)variance components. Following the estimation of the Cholesky model, we proceeded with fitting a series of nested models by fixing the parameters for A and/or C (co)variance components to zero and comparing the log-likelihood values of the restricted models with the non-restricted models. Overall, we tested an ACE, AE, CE, and an E model.

### Common Pathway Model

**Fig. 1:**
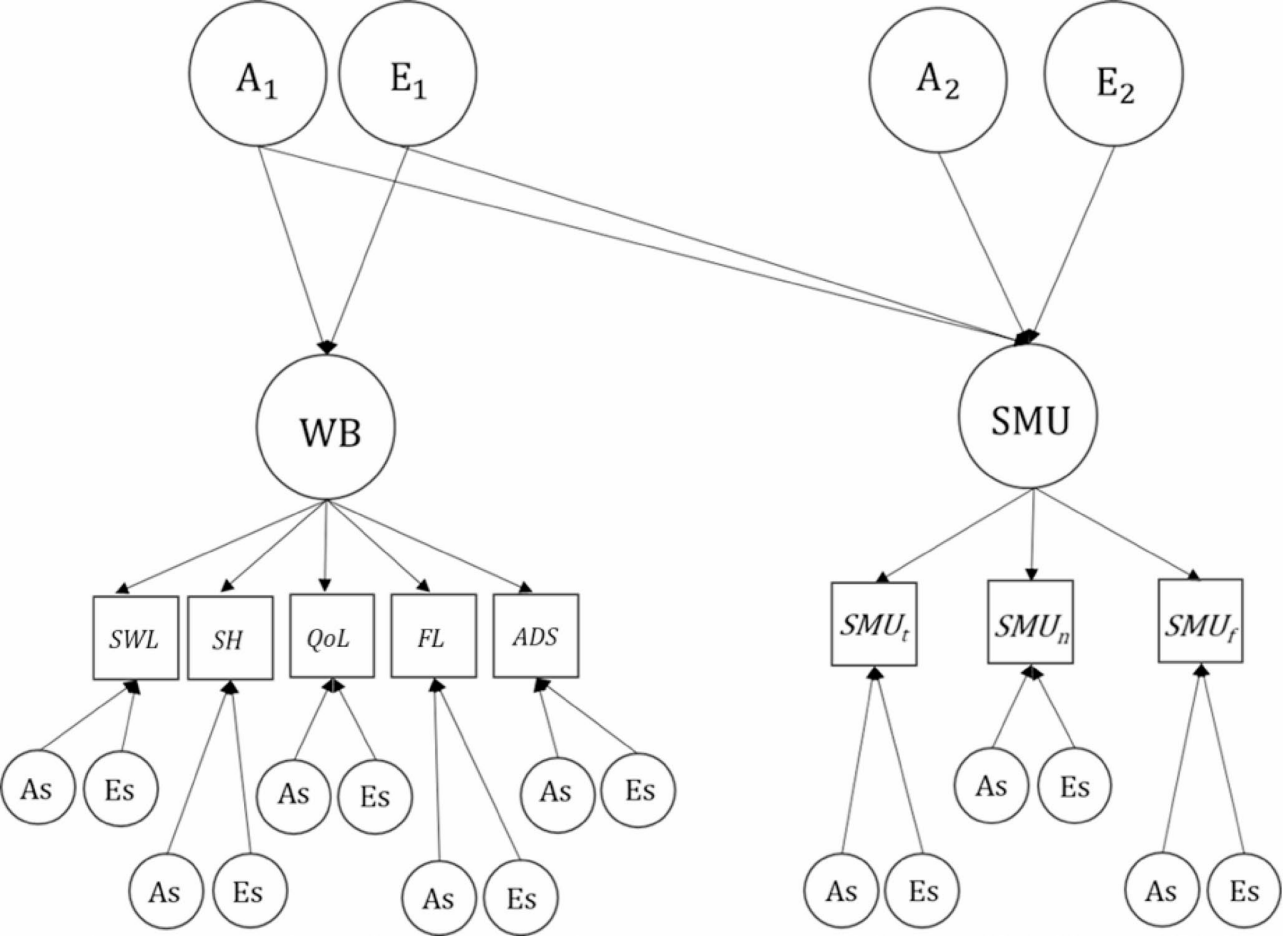
Two-factor common pathway model for a single twin (effects from covariates are not included for simplifying purposes). WB = wellbeing factor SMU, = social media use factor, A = additive genetic variance, E = environmental variance. As and Es refer to the phenotype-specific residual variances for A and E variances. *SWL* satisfaction with life, *SH* subjective happiness, *QoL* quality of life, *FL* flourishing, *ADS* anxious-depressive symptoms, *SMU*_*t*_ time spent (daily) on social media, *SMU*_*n*_ number of social media accounts, *SMU*_*f*_ frequency of posting on social media

In addition to the Cholesky model, the genetic and environmental effects can be modelled into common and specific factors in a common pathway (CP) model. More specifically, the CP model allows different phenotypes to be defined as indicators of a single or multiple latent factor(s), for which genetic and environmental variances can be estimated. In addition, variance components unique (i.e., independent of the common factor(s)) to each phenotype can be estimated (phenotype-specific/unique effects). In the CP model, a WB and an SMU common factor were specified. The WB factor is comprised of our WB wellbeing indicators and the ADS indicator (negatively weighted). The SMU factor is comprised of the three SMU variables (see Fig. 1). To make comparisons between the nested models, we used the chi-square difference tests.

## Results

### Descriptives and Phenotypic Correlations

**Fig. 2:**
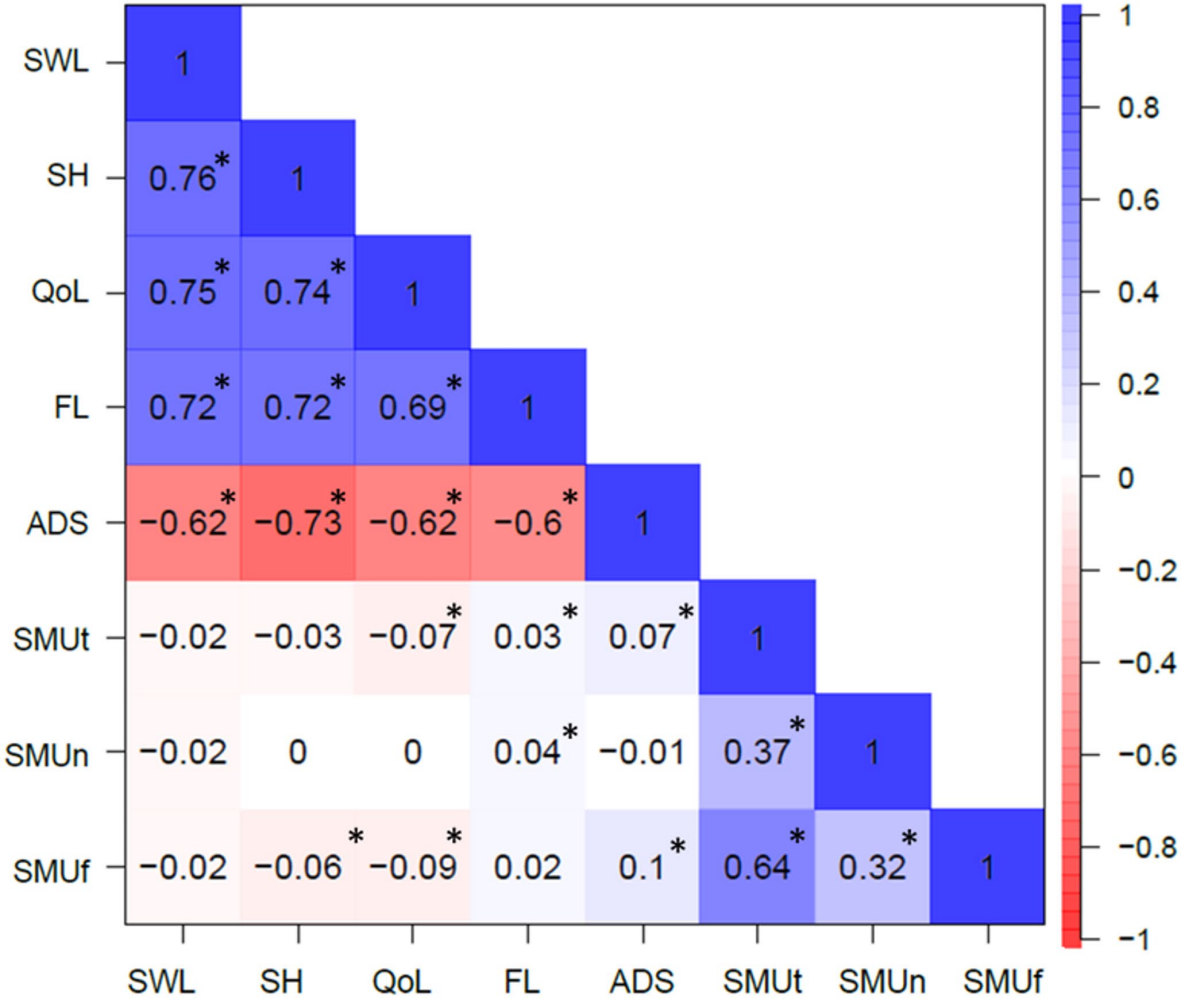
Phenotypic correlations among study variables. *significant correlation at alpha = 0.05. *SWL* satisfaction with life, *SH* subjective happiness, *QoL* quality of life, *FL* flourishing, *ADS* anxious-depressive symptoms, *SMU*_*t*_ time spent (daily) on social media, *SMU*_*n*_ number of social media accounts, *SMU*_*f*_ frequency of posting on social media

Table 1 shows the descriptive values and Fig. 2 shows the phenotypic correlations (with significance indicated at the alpha = 0.05 level). SMU_t_ and SMU_f_ were moderately correlated (*r* > 0.60), while SMU_n_ showed lower correlations with both SMU_t_ and SMU_f_ (*r* = 0.30–0.40). Approximately half of the phenotypic associations between WB/ADS and SMU (7 out of 15) were statistically significant at alpha = 0.05 but small, ranging between *r* = −0.09 and *r* = 0.10 (See Fig. 2). While the directions of the phenotypic correlations among SMU variables and among WB measures were consistent, they were not consistent between SMU and WB measures.

**Table 1:** Descriptives for the 8 phenotypic variables (N, M, SD, Range)

	*N*	M	SD	Range
SWL	5554	26.99	5.49	5–35
SHS	5541	22.07	4.77	4–28
QoL	5605	7.59	1.17	0–10
FL	5542	45.86	6.08	8–56
ADS	4207	5.68	6.06	0–33
SMU_t_	4295	8.6	2.74	5–26
SMU_n_	4732	2.66	1.54	0–6
SMU_f_	4699	2.24	1.07	1–5

*SWL:* Satisfaction with life, *SHS:* subjective happiness, *QoL:* quality of life, *FL:* flourishing, *ADS:* anxious-Depressive symptoms, *SMU*_*t*_: time spent (daily) on social media, *SMU*_*n*_: number of social media accounts, *SMU*_*f*_: frequency of posting on social media

### Covariates - Age/Sex and Assumption Tests

Age and sex had a significant effect on five and six of the eight variables, respectively. Higher age was positively associated with SHS (B = 0.035, SE = 0.004, *p* < 0.0001), QoL (B = 0.011, SE = 0.001, *p* < 0.0001) and negatively associated with SMU_t_ (B = −0.088, SE = 0.003, *p* < 0.0001) and SMU_f_ (B = −0.048, SE = 0.001, *p* < 0.0001). Females had lower SWL (B = −0.585, SE = 0.163, *p* < 0.001), SHS (B = −0.389, SE = 0.142, *p* < 0.010), QoL (B = −0.129, SE = 0.035, *p* < 0.001), higher level of ADS (B = 1.761, SE = 0.209, *p* < 0.0001), higher SMU_t_ (B = 0.656, SE = 0.092591125, *p* < 0.0001), and higher SMU_n_ (B = 0.220, SE = 0.035, *p* < 0.0001).

The chi-square difference tests showed the assumptions of equal means and variance across co-twins and zygosities were met (see Supplementary Table S2 for the exact model fitting results).

### Saturated Model

Table 2 contains the twin correlations and the cross-twin cross-trait correlations estimated in the saturated model. The consistently higher MZ (cross-twin, cross-trait) correlations than the DZ (cross-twin, cross-trait) correlations indicated that additive genetic influences explained part of the (co)variance for all phenotypes. There was no evidence for a genetic dominance effect, therefore we proceeded with estimating a Cholesky ACE model.

**Table 2:** Twin correlations and cross-twin cross-trait correlations

	SWL	SHS	QoL	FL	ADS	SMUt	SMUn	SMUf
SWL	0.42/0.26	0.20	0.23	0.20	−0.15	0.00	−0.02	−0.03
SHS	0.35	0.40/0.19	0.22	0.19	−0.16	−0.08	−0.06	−0.15
QoL	0.34	0.33	0.36/0.28	0.19	−0.18	−0.09	−0.08	−0.14
FL	0.34	0.33	0.31	0.36/0.19	−0.15	−0.01	−0.04	−0.05
ADS	−0.34	−0.40	−0.33	−0.32	0.49/0.25	0.10	0.04	0.15
SMU_t_	0.05	−0.01	−0.01	0.07	0.06	0.68/0.36	0.17	0.35
SMU_n_	0.01	0.00	0.03	0.06	−0.03	0.22	0.30/0.14	0.04
SMU_f_	0.03	−0.04	−0.01	0.06	0.05	0.48	0.18	0.53/0.45

MZ correlations below the diagonal and before the dash, DZ correlations above the diagonal and after the dash

*SWL:* satisfaction with life, *SHS:* subjective happiness, *QoL:* quality of life, *FL:* flourishing, *ADS:* anxious-depressive symptoms, *SMU*_*t*_: time spent (daily) on social media, *SMU*_*n*_: number of social media accounts, *SMU*_*f*_: frequency of posting on social media

### Cholesky Model

The Cholesky ACE model provided a better fit compared to the saturated model based on the chi-square difference test (*p*-value > 0.01) and the AIC values between the two models (174123.86 versus 173994.19; see Table 3). Further comparing the fit of a Cholesky AE model revealed the shared environmental (C) (co)variance components could be constrained to zero (*p*-value > 0.01, AIC 173955.43).

**Table 3:** Model fitting results for the multivariate models of wellbeing and social media use

	Baseline	Comparison	ep	−2LL	df	AIC	ΔLL	Δdf	*p*
1	Saturated model	–	304	173515.86	39,099	174123.86	–	–	–
2	Saturated model	**Cholesky ACE**	**140**	**173714.19**	**39,263**	**173994.19**	**198.33**	**164**	**0.035 (n.s.)**
3	Cholesky ACE	–	140	173714.19	39,263	173994.19			
4	Cholesky ACE	**Cholesky AE**	**104**	**173747.43**	**39,299**	**173955.43**	**33.24**	**36**	**0.60** **(n.s.)**
5	Cholesky ACE	Cholesky CE	104	173803.19	39,299	174011.19	89.01	36	0.000002
6	Cholesky ACE	Cholesky E	68	174742.04	39,335	174878.04	1027.85	72	0.000000
7	CP - ACE	–	73	174241.27	39,332	174387.27	–	–	–
8	CP - ACE	**CP - AE**	**62**	**174252.46**	**39,343**	**174376.46**	**11.19**	**11**	**0.43 (n.s.)**

*ep:* number estimated parameters, *− 2LL:* minus two times the log-likelihood, *df:* degrees of freedom, *AIC:* Akaike information criterion;, the best model was highlighted in bold for each comparison. *CP:* Common pathway model, *n.s.:* non-significant chi-square difference test result at alpha = 0.1

The unstandardized additive genetic (A) and unique environmental variances (E) based on the Cholesky AE model are shown in Supplementary Table S3. The unstandardized covariances for A and E are provided in Table S3. The standardized additive genetic (A) and unique environmental variances (E) are shown in Table 4. The standardized covariances for A and E are provided in Table 5. In general, additive genetic factors (A) explained a moderate share of the variance in WB/ADS (between 38% and 47%; see Table 4). Additive genetic influences on SMU traits ranged between 32% and 72%. The remaining variance is accounted for by non-shared environmental factors (E), which ranged between 53% and 62% for WB/ADS, and between 28% and 68% for the SMU variables.

**Table 4:** Standardized estimates for additive genetic effects (A) and unique environmental effects (E) from the best-fitting model (Cholesky AE)

	A (%)	E (%)
SWL	42 (37, 47)	58 (53, 63)
SH	39 (33, 44)	61 (56, 67)
QoL	39 (33, 44)	61 (56, 67)
FL	38 (32, 43)	62 (57, 68)
ADS	47 (41, 52)	53 (48, 59)
SMU_t_	72 (68, 75)	28 (25, 32)
SMU_n_	32 (25, 38)	68 (62, 75)
SMU_f_	54 (50, 59)	46 (41, 50)

*SWL:* satisfaction with life, *SH:* subjective happiness, *QoL:* quality of life, *FL:* flourishing, *ADS:* anxious-depressive symptoms, *SMU*_*t*_: time spent (daily) on social media, *SMU*_*n*_: number of social media accounts, *SMU*_*f*_: frequency of posting on social media

**Table 5:** Standardized variances and covariances in percentages for additive genetic effects (A) and unique environmental effects (E) (above diagonal) from the best fitting model (Cholesky AE)

	SWL	SH	QoL	FL	ADS	SMU_t_	SMU_*n*_	SMU_f_
SWL	39 (33, 44)/ 61 (56, 67)	55 (49,61)	54 (48,61)	53 (47,59)	51 (44,58)	NA	NA	NA
SH	45 (39,51)	39 (33, 44)/ 61 (56, 67)	54 (48,61)	52 (46,59)	51 (44,57)	NA	NA	NA
QoL	46 (39,52)	46 (39,52)	39 (33, 44)/ 61 (56, 67)	52 (46,59)	48 (41,55)	NA	NA	NA
FL	47 (41,54)	48 (41,54)	48 (41,54)	38 (32, 43)/ 62 (57, 68)	47 (40,55)	NA	NA	NA
ADS	49 (42,56)	50 (43,56)	52 (45,59)	53 (45,60)	47 (41, 52)/ 53 (48, 59)	NA	NA	NA
SMU_t_	NA	NA	NA	NA	NA	72 (68, 75)/ 28 (25, 32)	37 (28,48)	20 (16,25)
SMU_n_	NA	NA	NA	NA	NA	63 (53,73)	32 (25, 38)/ 68 (62, 75)	45 (34,58)
SMU_f_	NA	NA	NA	NA	NA	80 (75,84)	55 (43,66)	54 (50, 59)/ 46 (41, 50)

Standardized A variances are placed on the diagonal cells and within the diagonal cells, after the dash (‘/). Standardized E variances are also placed on the diagonal cells and within the diagonal cells, before the dash (‘/’), covariances are below diagonal. Standardized A covariances are placed in the cells that are below the diagonal, whereas the standardized E covariances are placed in the cells that are above the diagonal. NA represe *SWL* = satisfaction with life, *SH*: subjective happiness, *QoL*: quality of life, *FL*: flourishing, *ADS*::anxious-depressive symptoms,* SMU*_t_: time spent (daily) on social media, *SMU*_n_: number of social media accounts, *SMU*_f_: frequency of posting on social media. NA: CIs were unable to be estimated reliably given small phenotypic correlations. In some cases, unstandardized values for additive genetic and unique environmental covariances had the same signs (e.g., either both values were positive or negative). When the same signs for both values are not observed in the data, standardized genetic and environmental covariances result with percentages higher than 100 which cannot be interpreted meaningfully

Among the seven significant phenotypic correlations between WB/ADS and SMU variables, we only investigated the genetic and environmental influences on four of these associations^1^. The results indicated that phenotypic association between QoL with SMU_t_ and SMU_f_ (*r* = −0.07 and *r* = − 0.09 respectively) were largely determined by additive genetic factors (89% and 87%) in comparison to unique environmental factors (11% and 13%). Similarly, FL’s association with SMU_n_ (*r* = 0.04), and the correlation of ADS with SMU_t_ (*r* = 0.07) were largely determined by genetic factors (99% and 80% respectively), where the influence of environmental factors was much more limited (1% and 20%).

**Fig. 3:**
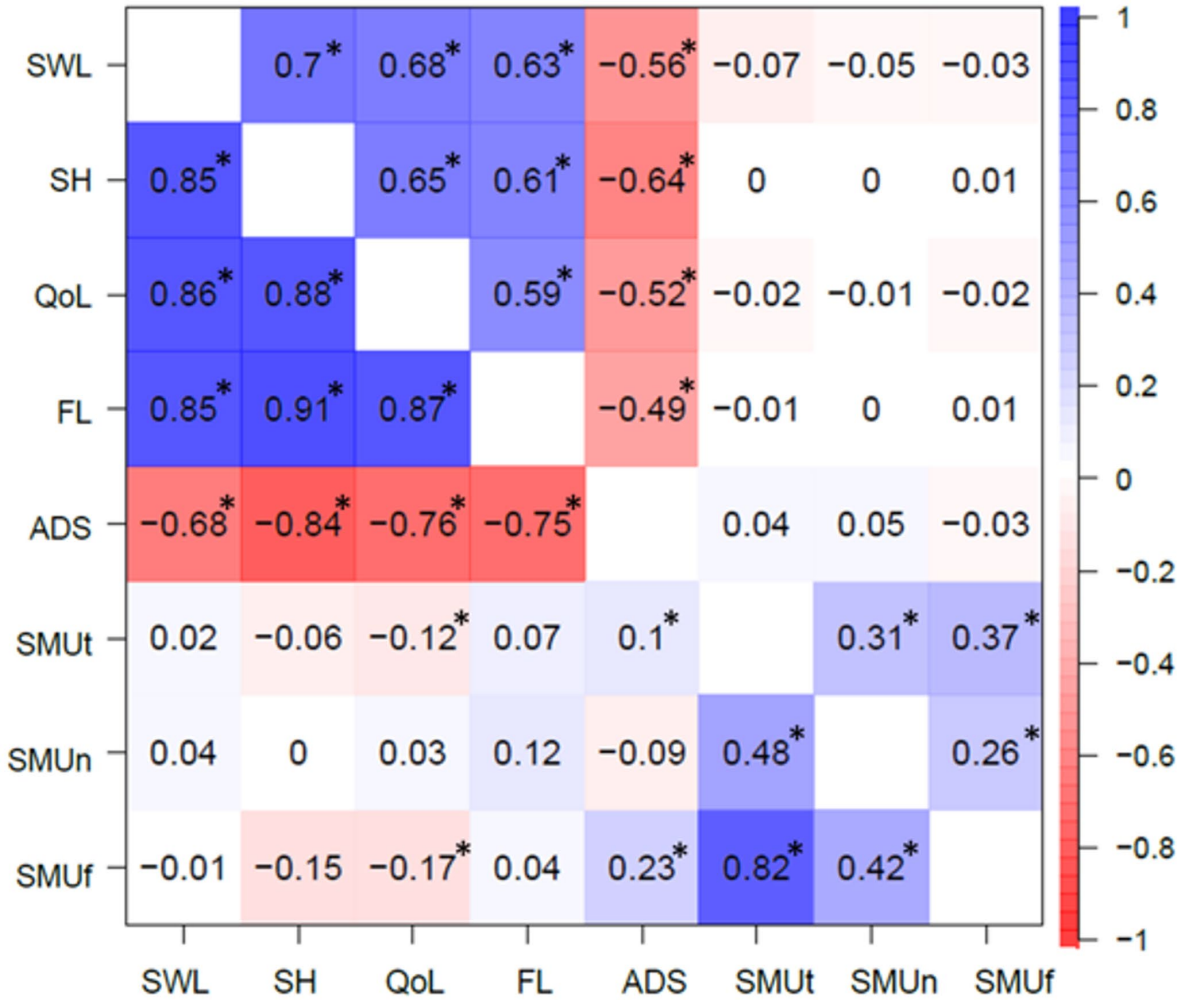
Genetic and environmental correlations (shown at the lower and upper diagonal of the table, respectively) among study variables. *significant correlation at alpha = 0.05. *SWL:* satisfaction with life, *SH:* subjective happiness, *QoL:* quality of life, *FL:* flourishing, *ADS:* anxious-depressive symptoms, *SMU*_*t*_: time spent (daily) on social media, *SMU*_*n*_: number of social media accounts, *SMU*_*f*_: frequency of posting on social media

Genetic and environmental correlations are shown in Fig. 3. The genetic correlations between the WB/ADS and SMU variables were small, and the environmental correlations were generally even smaller. In total, only four statistically significant genetic correlations were observed (at *p* < 0.05), while none of the environmental correlations reached statistical significance. Both QoL and ADS showed statistically significant genetic correlations with SMUt and SMUf but in an absolute sense these were relatively small (*r*_*g*_ = −0.12 and − 0.17 for QoL; *r*_*g*_ = 0.10 and 0.23 for ADS). These results suggest a potentially small but meaningful overlap between the genetic factors underlying WB/ADS variables and SMU.

### Common Factor Model

The common factor model with AE variance components provided a better fit than the model with all ACE components based on the chi-square differences test (*p*-value > 0.01) and the AIC values between the two models (174376.46 versus 174387.27, respectively). The Cholesky AE model provided a better fit than the common factor AE model (173955.43 versus 174376.46, respectively). Therefore, we refrain from discussing the common pathway model results (these can be found in Supplementary materials under Supplementary Figure F1–F3 and the section “Supplementary results from the common pathway model”).

## Discussion

The current study investigated the underlying sources of overlap between wellbeing (WB), anxious-depressive symptoms (ADS), and social media use (SMU) leveraging data from a large population-based twin sample. Consistent with the literature, we found small or no associations between WB/ADS and SMU variables. Social media use was heritable with estimates as high as 72% when measured through time spent daily on social media. The small but statistically significant associations between WB/ADS and SMU seemed to be due to overlapping genetic factors that influence both WB/ADS and SMU. The strong phenotypic relationship between time spent on social media and posting frequency also manifested in their genetic and environmental correlations (Fig. 2).

The phenotypic correlation between SMU_t_ and SMU_f_ (*r* = 0.64) was primarily driven by additive genetic factors (80%), and the remaining part of the correlation can be explained by non-shared environmental influences. On the contrary, SMU_n_’s phenotypic correlations with SMU_t_ (*r* = 0.37) and SMU_f_ (*r* = 0.32) were less strongly influenced by additive genetic factors (63% and 55%, respectively) but more by non-shared environmental influences (about 37% and 45%, respectively). In addition to these, we found a strong genetic correlation between SMU_t_ and SMU_f_ (*r* = 0.82), which indicated strong overlap in genetic influences on these two SMU variables. On the other hand, SMU_n_ had smaller genetic correlations with SMU_t_ and SMU_f_ (*r* = 0.48 and 0.42), indicating a smaller overlap with the other two SMU variables. We did not find strong environmental correlations among any of the SMU variables (*r* = ranging from 0.26 to 0.37) which indicated overlap in environmental influences were limited for these variables. These findings indicate that any intervention or prevention effort to limit the number of social media accounts will likely not generalize to time spent on social media and posting frequency. Alternatively, interventions to limit time spent on social media and posting frequency may have a greater chance of success due to their larger (genetic) overlap with each other, when the aim is to limit (problematic) amounts of social media use.

We found heritability estimates of 72% for time spent on social media (SMU_t_; 95% CI = 68–75%), 32% for number of social media accounts (SMU_n_; 25–38%), and 54% for social media use frequency (SMU_f_; 50–59%). These results align with previous findings on general internet and media use, which reported heritability estimates ranging from 24 to 67% (Ayorech et al. 2017, 2023; Long et al. 2015; York 2017), and 21–66% for problematic internet/media use (Ayorech et al. 2023; Deryakulu & Ursavas, 2014; Hahn et al. 2017; Li et al. 2014). Among these, Ayorech et al. (2017) is particularly relevant due to its focus on social media, specifically Facebook use, in a sample of 16-year-old twins. Facebook use was operationalized as a composite of three measures: how long participants had a Facebook account, how often they checked updates, and the number of Facebook friends they had. This composite measure for social media use was found to be 24% heritable (CI = 17–32%).

In contrast, our study measured social media use across multiple widely used platforms which were Facebook, Instagram, Snapchat, Twitter, and LinkedIn, and included a broader age range (M = 35.10 years, SD = 14.93, range = 16–89). Notably, we observed heritability estimates as high as 72% for time spent on social media (SMUt). Variability in heritability estimates across studies may stem from differences in how social media use was operationalized, as well as variations in the age composition of the samples. Future research could benefit from examining an even wider range of platforms and more diverse age groups. While our aim in the present study was to investigate associations across a broad age range to enhance generalizability, we acknowledge that developmental factors such as self-control and risk-taking may moderate these associations. In our study, we controlled for the net effects of age (via including it as a covariate), nevertheless, age moderation could be investigated in future studies, though potentially requiring bigger sample sizes than ours. Overall, the mentioned development factors can be also more meaningfully explored in future work focusing on specific age subgroups (Boyer 2006; Duckworth et al. 2014). Furthermore, given the adult nature of our sample, it is also possible that occupational demands influence social media use, as some professions require more frequent or platform-specific engagement. Future studies would benefit from incorporating occupational context to better understand such differences.

In general, our findings for the heritability of SMU showed that individuals may have different genetic liabilities which may result in very diverse social media use habits. This information can be utilized to explain differences in social media use both across different individuals but also across different families. Relatedly, the social media usage patterns of parents can be used to understand the social media use of the children and adolescents, since parents’ and offspring’s social media behaviour may be subject to shared genetic influences. Information from the parents can be particularly useful for setting realistic goals in terms of determining the efficacy of interventions in which the aim is to decrease excessive amount of social media use, also referred to as “addictive social media use” (Andreassen et al. 2017). In addition, especially in adolescent target groups, interventions that primarily focus on leveraging within-family-based support to control addictive social media use might be less effective, as familial genetic predisposition might play a role in developing (problematic) patterns of social media use.

In the present study, 7 out of 15 phenotypic correlations between WB/ADS and SMU were statistically significant, yet small (*r*s between − 0.09 and 0.10. These results align with meta-review studies for wellbeing and illbeing variables (Orben 2020; Parry et al. 2022; Valkenburg 2022) which have reported similar small or null associations between WB/ADS and SMU, underlining the potentially complex nature of the associations between these variables. Although strong claims are often made on the negative effects of social media use on wellbeing (e.g., Haidt, 2024), these are not substantiated in the current study.

Looking into these associations in more detail, we found that different WB measures manifested different correlations with SMU depending on the specific WB measure. Specifically, flourishing was positively associated with having more social media accounts (SMU_n_) and spending more time on social media (SMU_t_). The other WB measures were not related to having more social media accounts. Moreover, the signs of the associations between flourishing and the SMU variables were positive, whereas the other WB variables related to the SMU variables negatively. Forinstance, higher hedonic wellbeing levels negatively correlated with spending more time on social media (SMU_t_) and more frequently posting on social media (SMU_f_).

As the associations between WB/ADS and SMU variables were very small, they require caution when interpreting them. Nonetheless, it could be speculated that individuals with higher flourishing levels combine the use of more social media channels to utilize the unique advantages or the properties that each platform may offer (for instance, one platform can be better at following work related updates). The same individuals may also spend more time on social media, yet they do not post more frequently. This may indicate that higher flourishing might be associated with a more passive way of engaging with social media as opposed to more active ways such as posting. This is in line with the evidence that adolescents who flourish may spend more time on social media, but do not report themselves as ‘oversharing their activities’ compared to the general, ‘neither flourishing nor languishing’ group of users (Law et al., 2020). In contrast, lower hedonic wellbeing levels may be related to spending more time on social media and posting more frequently without combining the use of multiple social media platforms. This finding is partly supported by previous evidence suggesting that oversharing on social media and dysfunctional amounts of social media use to be related to lower wellbeing levels in adolescents (Shabahang et al. 2024). Given the small overall correlation pattern, our interpretations of the present results have to be verified by future research.

Overall, in the present study, given the (very) small phenotypic correlations between WB/ADS and SMU the covariance estimates for the genetic and environmental influences mentioned above should be still interpreted with care. For instance, the phenotypic correlations between WB/ADS and SMU (which were generally smaller than absolute *r* = 0.10) that were statistically significant were largely determined by additive genetic factors (between 80 and 90%). Although this was the case, whenever the same signs for both values are not observed in the data, standardized genetic and environmental covariances results with percentages higher than 100 which cannot be interpreted meaningfully. Furthermore, the small phenotypic correlations often yielded inaccurate and problematic confidence intervals estimates for the standardized A and E covariances (we indicated such cases as NA in Table 5). The full list of the unstandardized A and E covariances estimates and the associated CI values are shown in Supplemental Materials under Table S4.

Furthermore, we found that both quality of life and ADS had statistically significant genetic correlations with SMU_t_ and SMU_f_ (*r*_*g*_ = −0.12 and − 0.17 for quality of life; *r*_*g*_ = 0.10 and 0.23 for ADS). No significant environmental correlations were found between WB/ADS and SMU variables which were also considerably small in magnitude as well (the highest value in absolute terms was *r*_*e*_ = − 0.07). These findings are in line with previous research similarly reporting small phenotypic associations between general media use and prosocial behavior (e.g., *r* = 0.19), which were also predominantly explained by additive genetic influences (about 88%) and had statistically significant genetic correlations (*r*_*g*_ = 0.37) (Ayorech et al. 2023). Different from the present study’s results, the same previous work has reported statistically significant environmental correlation between general media use and prosocial behavior, though such an estimate was also very small in magnitude similar to ours (i.e., *r*_*e*_ = 0.08). All in all, the present study extends these previous findings by specifically examining social media use across multiple platforms and by incorporating a broader range of both hedonic and eudaimonic wellbeing indicators and support that the association between WB/ADS and SMU may be (partly) due to underlying genetic influences. Additionally, the inclusion of a more diverse age range in the present study’s sample suggests that the influence of additive genetic factors on WB/ADS and SMU may not be confined to a particular developmental stage. Overall, researchers may also consider not only the phenotypic associations observed between these variables but also the additional nuance provided by genetically informed designs such as the one employed in the present study.

### Limitations

In the present study, we had certain limitations. First, the present sample size did not allow for testing quantitative and qualitative sex differences. However, we included sex as a covariate to account for its influence on mean levels of WB/ADS and SMU. Second, the data from the present study were cross-sectional, making it difficult to make claims regarding the direction of causation between the variables. That said, we acknowledge that Direction of Causation (DoC) models can, in principle, be applied to cross-sectional data and may yield valuable insights. However, these models rely on several assumptions and prerequisites that are not always easily satisfied (for example, differing genetic architectures across traits and the need to model shared variance using common latent factors). In our case, the common pathway model provided a suboptimal fit to the data, suggesting that this approach may not be ideal here. Additionally, the two phenotypes under study (wellbeing/mental health and social media use) showed only a weak phenotypic correlation, further limiting the utility of DoC models for our dataset. Nonetheless, we agree that future research could benefit from exploring this approach, particularly in samples better suited to its assumptions. Fourth, our study employed the classical twin design, which has a series of assumptions (such as not modelling existing gene-environment correlations) (Derks et al., 2004; Kendler et al. 1993). In the present study, we tested for equal mean and variances between twin siblings, and MZ and DZ twins for each of the phenotypes. However, we did not test for random mating, the equal environment assumption, or whether there were any gene-environment correlations, and gene-environment interactions present in the data. Among these unmodeled potential interplays, gene-environment interactions and gene-environment correlations can cause the overestimation of A and E variances, respectively (Little 2013). Moreover, the presence of assortative mating, if not modelled, causes underestimated A and overestimated C variances (Little 2013). Although we did not test the equal environments assumption, it usually holds for many phenotypes (Willoughby et al. 2023). In addition, the current study design cannot determine whether there is a causal relationship between social media use and wellbeing; it can only highlight that their association may be influenced by shared genetic factors. Moreover, we also cannot rule out whether the direction of the relationship between the two variables goes from wellbeing/mental health to social media use, the other way around or in both ways. For insights on the direction of causality, future studies would need to employ designs specifically suited for causality questions such as experience sampling methods combined with genetic information (De Vries and Bartels 2025). Furthermore, our sample was from the Netherlands; therefore, the heritability estimates found for each phenotype may differ in other cultures, as it was found for other behavioural variables such as personality (Jang et al. 2006). Future research should replicate these findings in samples from other cultures and populations.

Given the ratio of incomplete twin pairs to complete twin pairs, we additionally conducted t-tests on the average values observed for eachgroup across the phenotypes. We calculated Cohen’s d estimates with 95% lower and upper Confidence Intervals. Results indicated some statistically significant differences (*p*-values smaller than 0.05), though all of the calculated effect sizes remained below 0.20, which can be interpreted as negligible (Cohen 1988). Nevertheless, our results should be interpreted in light of this information (the full results can be found in Supplementary Table S1), and future studies including more complete twin pairs is warranted. Lastly, the distribution of the variables ADS and SMU_t_ showed signs of being positively skewed (the lowest scores were the most frequently present in the dataset). As a part of our pre-registered post-hoc analysis plan, we have repeated our main analyses, with transformed values for ADS and SMU_t_ to make them more normally distributed. The results revealed that our heritability estimates did not significantly change as the confidence intervals between the original and post-hoc heritability estimates overlapped with one another (see Supplementary Table S6 and S7).

### Concluding Remarks

The present study employed a classical twin design based on a population-based sample to explore the underlying sources of overlap between wellbeing, anxious-depressive symptoms, and social media use. Our results confirmed the small associations between social media use and wellbeing/anxious-depressive symptoms in the literature (Appel et al. 2020; Valkenburg 2022, ), and suggested these associations to be partly driven by overlapping genetic factors. Further, we showed general social media use to be heritable as high as 72%. Given our findings, we encourage researchers and experts to consider more personalized approaches when considering the association between wellbeing and social media use intensity, as well as when understanding the reasons for individuals’ use of social media use itself.

## Electronic Supplementary Material

Below is the link to the electronic supplementary material.

Supplementary Material 1

## Data Availability

The data from the Netherlands Twin Register can be requested from the Netherlands Twin Register (https://ntr-data-request.psy.vu.nl/) after completing the requirements of the data access committee.

## Footnotes

1 This was because only in 4 of these 7 cases, additive genetic and environmental covariances had the same signs (e.g., either both values were positive or negative). When the same signs for both values are not observed in the data, genetic and environmental covariances cannot be interpreted in a meaningful way.
