# Supplementary material for: The Association Between Frequency of Social Media Use, Wellbeing, and Depressive Symptoms: Disentangling Genetic and Environmental Factors
Source: Behav Genet. 2025 Jun 21;55(4):255–69. doi: 10.1007/s10519-025-10224-2 (PMC12325487; doi:10.1007/s10519-025-10224-2)
Supplement: Supplementary file 1 — Supplementary Material 1 [file 10519_2025_10224_MOESM1_ESM.docx]

**Supplementary Material**

**Supplementary Table S1.** Welch Two Sample t-test results and Cohen’s d estimates

| **Variable** | **Mean complete** | **Mean incomplete** | ***p*-value** | **Cohen’s d** | **Lower CI** | **Upper**  **CI** | **Item range** |  |
| --- | --- | --- | --- | --- | --- | --- | --- | --- |
| SWL | 27.348 | 26.744 | 0 | 0.114 | 0.046 | 0.183 | 5-35 |  |
| SHS | 22.264 | 21.917 | 0.016 | 0.076 | 0.007 | 0.144 | 4-28 |  |
| QoL | 7.656 | 7.54 | 0.001 | 0.103 | 0.034 | 0.171 | 0-10 |  |
| FL | 46.011 | 45.759 | 0.167 | 0.043 | -0.025 | 0.112 | 8-56 |  |
| ADS | 5.575 | 5.761 | 0.398 | -0.032 | -0.113 | 0.05 | 0-33 |  |
| SMU_t_ | 8.214 | 8.654 | 0 | -0.154 | -0.235 | -0.073 | 5-26 |  |
| SMU_n_ | 2.533 | 2.775 | 0 | -0.164 | -0.238 | -0.089 | 0-6 |  |
| SMU_f_ | 2.217 | 2.266 | 0.177 | -0.046 | -0.122 | 0.029 | 1-5 |  |
| *Note.* SWL = satisfaction with life, SHS = subjective happiness, QoL = quality of life, FL = flourishing, ADS = anxious-depressive symptoms, SMU_t_ = time spent (daily) on social media, SMU_n_ = number of social media accounts owned, SMU_f_ = posting on social media. CI = 95% Confidence intervals for Cohen’s d estimates. | | | | | | | | |

**Supplementary Table S2.** Classical twin model assumption tests for each phenotype together with inspection of the covariates - age and sex

| SWL |  |  |  |  |  |  |  |  |  |
| --- | --- | --- | --- | --- | --- | --- | --- | --- | --- |
| **Baseline** | **Comparison** | **ep** | **-2LL** | **df** | **AIC** | **ΔLL** | **Δdf** | ***p*** |  |
| Sat. model | - | 12 | 33818.47 | 5430.00 | 33842.47 | - | - | - |  |
|  | age = 0 | 11 | 33821.94 | 5431.00 | 33843.94 | 3.48 | 1.00 | 0.06 |  |
|  | sex = 0 | 11 | 33818.53 | 5431.00 | 33840.53 | 0.06 | 1.00 | 0.80 |  |
|  | equal M twin1 vs. twin2; age = 0, sex = 0 | 8 | 33822.25 | 5434.00 | 33838.25 | 3.78 | 4.00 | 0.44 |  |
|  | equal V twin1 vs. twin2; age = 0, sex = 0 | 8 | 33825.27 | 5434.00 | 33841.27 | 6.80 | 4.00 | 0.15 |  |
|  | **equal M and V twin1 vs. twin2 and MZ vs. DZ; age = 0, sex = 0** | **4** | **33829.68** | **5438.00** | **33837.68** | **11.22** | **8.00** | **0.19** |  |
|  |  |  |  |  |  |  |  |  |  |
| QoL |  |  |  |  |  |  |  |  |  |
| **Baseline** | **Comparison** | **ep** | **-2LL** | **df** | **AIC** | **ΔLL** | **Δdf** | ***p*** |  |
| Sat. model | - | 12 | 17074.42 | 5474.00 | 17098.42 | - | - | - |  |
|  | age = 0 | 11 | 17163.59 | 5475.00 | 17185.59 | 89.17 | 1.00 | 0.00 |  |
|  | sex = 0 | 11 | 17074.47 | 5475.00 | 17096.47 | 0.04 | 1.00 | 0.84 |  |
|  | equal M twin1 vs. twin2 | 10 | 17074.90 | 5476.00 | 17094.90 | 0.48 | 2.00 | 0.79 |  |
|  | equal V twin1 vs. twin2 | 10 | 17075.24 | 5476.00 | 17095.24 | 0.82 | 2.00 | 0.67 |  |
|  | **equal M and V twin1 vs. twin2 and MZ vs. DZ** | **6** | **17077.23** | **5480.00** | **17089.23** | **2.81** | **6.00** | **0.83** |  |
|  |  |  |  |  |  |  |  |  |  |
| ADS |  |  |  |  |  |  |  |  |  |
| **Baseline** | **Comparison** | **ep** | **-2LL** | **df** | **AIC** | **ΔLL** | **Δdf** | ***p*** |  |
| Sat. model | - | 12 | 26204.65 | 4102.00 | 26228.65 | - | - | - |  |
|  | covariate: age | 11 | 26369.95 | 4103.00 | 26391.95 | 165.31 | 1.00 | 0.00 |  |
|  | covariate: sex | 11 | 26212.73 | 4103.00 | 26234.73 | 8.08 | 1.00 | 0.00 |  |
|  | equal M twin1 vs. twin2 | 10 | 26209.77 | 4104.00 | 26229.77 | 5.12 | 2.00 | 0.08 |  |
|  | equal V twin1 vs. twin2 | 10 | 26206.60 | 4104.00 | 26226.60 | 1.95 | 2.00 | 0.38 |  |
|  | **equal M and V twin1 vs. twin2 and MZ vs. DZ** | **6** | **26212.69** | **4108.00** | **26224.69** | **8.04** | **6.00** | **0.24** |  |
|  |  |  |  |  |  |  |  |  |  |
| FL |  |  |  |  |  |  |  |  |  |
| **Baseline** | **Comparison** | **Ep** | **-2LL** | **df** | **AIC** | **ΔLL** | **Δdf** | ***p*** |  |
| Sat. model | - | 12 | 34815.78 | 5413.00 | 34839.78 | - | - | - |  |
|  | age = 0 | 11 | 34815.81 | 5414.00 | 34837.81 | 0.03 | 1.00 | 0.86 |  |
|  | sex = 0 | 11 | 34816.71 | 5414.00 | 34838.71 | 0.93 | 1.00 | 0.33 |  |
|  | equal M twin1 vs. twin2 | 10 | 34819.37 | 5415.00 | 34839.37 | 3.58 | 2.00 | 0.17 |  |
|  | equal V twin1 vs. twin2 | 10 | 34819.15 | 5415.00 | 34839.15 | 3.37 | 2.00 | 0.19 |  |
|  | **equal M and V twin1 vs. twin2 and MZ vs. DZ** | **6** | **34823.48** | **5419.00** | **34835.48** | **7.70** | **6.00** | **0.26** |  |
|  |  |  |  |  |  |  |  |  |  |
| SH |  |  |  |  |  |  |  |  |  |
| **Baseline** | **Comparison** | **ep** | **-2LL** | **df** | **AIC** | **ΔLL** | **Δdf** | ***p*** |  |
| Sat. model | - | 12 | 32222.36 | 5421.00 | 32246.36 | - | - | - |  |
|  | covariate: age | 11 | 32276.49 | 5422.00 | 32298.49 | 54.13 | 1.00 | 0.00 |  |
|  | covariate: sex | 11 | 32222.36 | 5422.00 | 32244.36 | 0.00 | 1.00 | 0.96 |  |
|  | equal M twin1 vs. twin2; sex = 0 | 9 | 32223.38 | 5424.00 | 32241.38 | 1.03 | 3.00 | 0.79 |  |
|  | equal V twin1 vs. twin2; sex = 0 | 9 | 32223.24 | 5424.00 | 32241.24 | 0.88 | 3.00 | 0.83 |  |
|  | **equal M and V twin1 vs. twin2 and MZ vs. DZ; sex = 0** | **5** | **32225.67** | **5428.00** | **32235.67** | **3.31** | **7.00** | **0.85** |  |
|  |  |  |  |  |  |  |  |  |  |
| SMU_t_ |  |  |  |  |  |  |  |  |  |
| **Baseline** | **Comparison** | **ep** | **-2LL** | **df** | **AIC** | **ΔLL** | **Δdf** | ***p*** |  |
| Sat. model | - | 12 | 19124.89 | 4201.00 | 19148.89 | - | - | - |  |
|  | age = 0 | 11 | 20028.18 | 4202.00 | 20050.18 | 903.30 | 1.00 | 0.00 |  |
|  | sex = 0 | 11 | 19150.80 | 4202.00 | 19172.80 | 25.92 | 1.00 | 0.00 |  |
|  | equal M twin1 vs. twin2 | 10 | 19127.23 | 4203.00 | 19147.23 | 2.34 | 2.00 | 0.31 |  |
|  | equal V twin1 vs. twin2 | 10 | 19126.59 | 4203.00 | 19146.59 | 1.70 | 2.00 | 0.43 |  |
|  | **equal M and V twin1 vs. twin2 and MZ vs. DZ** | **6** | **19133.61** | **4207.00** | **19145.61** | **8.72** | **6.00** | **0.19** |  |
|  |  |  |  |  |  |  |  |  |  |
| SMU_n_ |  |  |  |  |  |  |  |  |  |
| **Baseline** | **Comparison** | **ep** | **-2LL** | **df** | **AIC** | **ΔLL** | **Δdf** | ***p*** |  |
| Sat. model | - | 12 | 13534.05 | 4583.00 | 13558.05 | - | - | - |  |
|  | age = 0 | 11 | 13536.98 | 4584.00 | 13558.98 | 2.94 | 1.00 | 0.09 |  |
|  | sex = 0 | 11 | 13554.45 | 4584.00 | 13576.45 | 20.41 | 1.00 | 0.00 |  |
|  | equal M twin1 vs. twin2; age = 0 | 9 | 13538.03 | 4586.00 | 13556.03 | 3.98 | 3.00 | 0.26 |  |
|  | equal V twin1 vs. twin2; age = 0 | 9 | 13537.99 | 4586.00 | 13555.99 | 3.94 | 3.00 | 0.27 |  |
|  | **equal M and V twin1 vs. twin2 and MZ vs. DZ; age = 0** | **5** | **13543.81** | **4590.00** | **13553.81** | **9.76** | **7.00** | **0.20** |  |
|  |  |  |  |  |  |  |  |  |  |
| SMU_f_ |  |  |  |  |  |  |  |  |  |
| **Baseline** | **Comparison** | **ep** | **-2LL** | **df** | **AIC** | **ΔLL** | **Δdf** | ***p*** |  |
| Sat. model | - | 12 | 15779.32 | 4616.00 | 15803.32 | - | - | - |  |
|  | age = 0 | 11 | 16811.96 | 4617.00 | 16833.96 | 1032.63 | 1.00 | 0.00 |  |
|  | sex = 0 | 11 | 15779.62 | 4617.00 | 15801.62 | 0.30 | 1.00 | 0.59 |  |
|  | equal M twin1 vs. twin2; sex = 0 | 9 | 15781.12 | 4619.00 | 15799.12 | 1.80 | 3.00 | 0.61 |  |
|  | equal V twin1 vs. twin2; sex = 0 | 9 | 15790.30 | 4619.00 | 15808.30 | 10.98 | 3.00 | 0.01 |  |
|  | **equal M = V for siblings and MZ/DZ; sex = 0** | **5** | **15792.88** | **4623.00** | **15802.88** | **13.56** | **7.00** | **0.06** |  |
|  |  |  |  |  |  |  |  |  |  |
| *Note.* Sat. model = saturated model, M = mean, V = variance, ep = number estimated parameters, − 2LL = minus two times the log-likelihood, df = degrees of freedom, AIC = akaike information criterion; best-fitting model in bold letters, CP = common pathway model, n.s. = non-significant chi-square difference test result at alpha = 0.1. SWL = satisfaction with life, SH = subjective happiness, QoL = quality of life, FL = flourishing, ADS = anxious-depressive symptoms, SMU_t_ = time spent (daily) on social media, SMU_n_ = number of social media accounts owned, SMU_f_ = posting on social media. | | | | | | | | |  |
|  |  |  |  |  |  |  |  |  |  |
|  |  |  |  |  |  |  |  |  |  |
|  |  |  |  |  |  |  |  |  |  |
|  |  |  |  |  |  |  |  |  |  |
|  |  |  |  |  |  |  |  |  |  |

**Supplementary Table S3.** Unstandardized estimates for additive genetic effects (A) and unique environmental effects (E) from the best fitting model (Cholesky AE).

|  | **A** | **E** | **Total Variance** |
| --- | --- | --- | --- |
| SWL | 12.72 (10.92,14.48) | 17.40 (15.88, 19.09) | 30.12 |
| SHS | 8.81 (7.40, 10.20) | 13.93 (12.7, 15.29) | 22.74 |
| QoL | 0.53 (0.44, 0.61) | 0.84 (0.76, 0.92) | 84.53 |
| FL | 13.87 (11.62, 16.07) | 22.74 (20.77, 24.91) | 36.61 |
| ADS | 16.86 (14.58, 19.09) | 18.96 (17.11, 21.04) | 35.82 |
| SMU_t_ | 5.33 (4.92, 5.74) | 2.10 (1.86, 2.37) | 7.43 |
| SMU_n_ | 0.36 (0.28, 0.43) | 0.77 (0.70, 0.85) | 30.12 |
| SMU_f_ | 1.28 (1.16, 1.41) | 1.08 (0.98, 1.19) | 22.74 |

*Note.* SWL = satisfaction with life, SHS = subjective happiness, QoL = quality of life, FL = flourishing, ADS = anxious-depressive symptoms, SMU_t_ = time spent (daily) on social media, SMU_n_ = number of social media accounts owned, SMU_f_ = posting on social media.

**Supplementary Table S4.** Unstandardized covariance estimates for additive genetic effects (A) (below diagonal) and unique environmental effects (E) (above diagonal) from the best fitting model (Cholesky AE).

|  | SWL | SH | QoL | FL | ADS | SMU_t_ | SMU_n_ | SMU_f_ |
| --- | --- | --- | --- | --- | --- | --- | --- | --- |
| SWL | - | 10.85 (9.66,12.16) | 2.61 (2.32,2.93) | 12.52 (11.06,14.12) | -10.25 (-11.77, -8.85) | -0.4 (-0.89,0.07) | -0.19 (-0.44,0.05) | -0.14 (-0.44,0.15) |
| SH | 8.95 (7.55,10.31) | - | 2.23 (1.98,2.52) | 10.89 (9.58,12.33) | -10.47 (-11.88, -9.18) | 0.01 (-0.42,0.44) | 0.01 (-0.21,0.23) | 0.05 (-0.21,0.31) |
| QoL | 2.22 (1.88,2.55) | 1.89 (1.59,2.18) | - | 2.57 (2.25,2.92) | -2.08 (-2.41, -1.78) | -0.02 (-0.13,0.08) | -0.01 (-0.06,0.04) | -0.02 (-0.09,0.04) |
| FL | 11.27 (9.56,12.95) | 10.01 (8.47,11.51) | 2.34 (1.97,2.7) | - | -10.25 (-11.96, -8.68) | -0.1 (-0.65,0.45) | 0 (-0.27,0.28) | 0.06 (-0.27,0.39) |
| ADS | -9.97 (-11.61, -8.31) | -10.26 (-11.76, -8.73) | -2.27 (-2.63, -1.91) | -11.39 (-13.21, -9.54) | - | -0.1 (-0.65,0.45) | 0 (-0.27,0.28) | 0.06 (-0.27,0.39) |
| SMU_t_ | 0.15 (-0.47,0.76) | -0.4 (-0.95,0.14) | -0.2 (-0.34, -0.06) | 0.64 (-0.05,1.33) | 0.9 (0.22,1.59) | - | 0.39 (0.29,0.5) | 0.55 (0.43,0.68) |
| SMU_n_ | 0.08 (-0.18,0.34) | 0.01 (-0.22,0.24) | 0.01 (-0.04,0.07) | 0.28 (-0.01,0.56) | -0.22 (-0.53,0.08) | 0.67 (0.54,0.8) | - | 0.24 (0.18,0.3) |
| SMU_f_ | -0.03 (-0.37,0.31) | -0.51 (-0.81, -0.2) | -0.14 (-0.21, -0.06) | 0.17 (-0.21,0.55) | 1.08 (0.69,1.47) | 2.14 (1.96,2.33) | 0.29 (0.22,0.36) | - |

*Note.* SWL = satisfaction with life, SH = subjective happiness, QoL = quality of life, FL = flourishing, ADS = anxious-depressive symptoms, SMU_t_ = time spent (daily) on social media, SMU_n_ = number of social media accounts owned, SMU_f_ = posting on social media.

**Supplementary Table S5.** Mean and median estimates for the responses provided for social media use motivations by the participants in the present study.

| General question: For what reasons do you use social media? 1 – 7 (strongly disagree – strongly agree) | | |
| --- | --- | --- |
| **Item** | **Mean (CI % 95)** | **Median** |
| To keep in touch with friends and family | 2.96 (2.94, 2.99) | 3.00 |
| To plan events | 1.99 (1.97, 2.01) | 1.99 |
| For buying and selling | 1.87 (1.85, 1.88) | 1.86 |
| Inspiration | 2.67 (2.64, 2.69) | 2.67 |
| News | 3.11 (3.08, 3.13) | 3.11 |
| Dating | 1.16 (1.15, 1.17) | 1.16 |

**Supplementary Table S6**. Standardized additive genetic variance components (SA) and standardized non-shared environmental variance components (SE) from the best fitting model (Cholesky AE), recalculated with log10 transformed ADS and SMU_t_ scores, as a part of our post-hoc sensitivity analysis.

|  | **A (%)** | **E (%)** |
| --- | --- | --- |
| SWL | 42 (37, 47) | 58 (53, 64) |
| SH | 39 (33, 44) | 61 (56, 67) |
| CL | 38 (32, 44) | 62 (56, 67) |
| FL | 38 (32, 43) | 62 (57, 68) |
| ADS | 42 (35, 48) | 58 (52, 65) |
| SMU_t_ | 72 (68, 74) | 28 (26, 32) |
| SMU_n_ | 31 (25, 37) | 69 (63, NA) |
| SMU_f_ | 54 (49, 58) | 46 (42, 51) |

*Note.* SWL = satisfaction with life, SH = subjective happiness, QoL = quality of life, FL = flourishing, ADS = anxious-depressive symptoms, SMU_t_ = time spent (daily) on social media, SMU_n_ = number of social media accounts owned, SMU_f_ = posting on social media.

**Supplementary Figure F1**. Unstandardized two-factor common pathway model for a single twin (effects from covariates are not included for simplifying purposes)


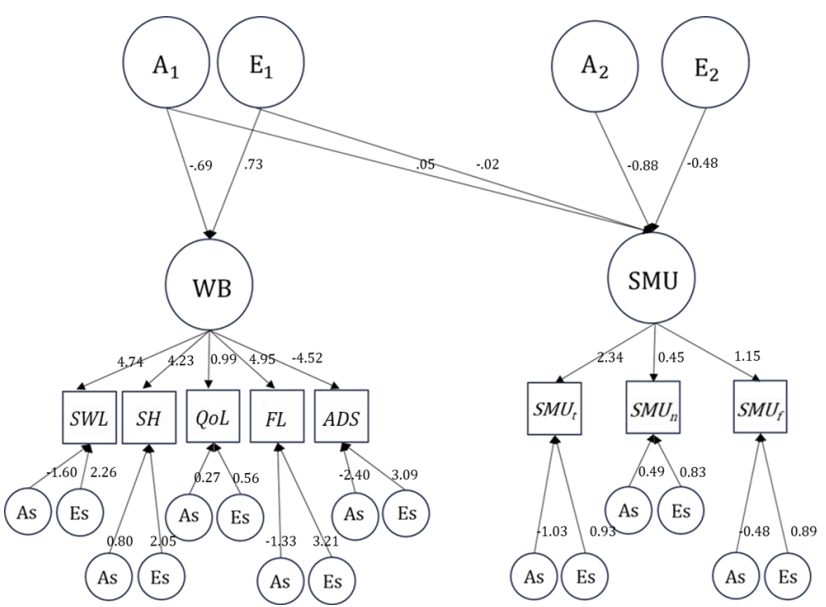


*Note.* WB = wellbeing factor SMU, = social media use factor, A = additive genetic variance, E =

environmental variance. As and Es refer to the phenotype-specific residual variances for A and E

variances. SWL = satisfaction with life, SH = subjective happiness, QoL = quality of life, FL =

flourishing, ADS = anxious-depressive symptoms, SMUt = time spent (daily) on social media, SMUn

= number of social media accounts, SMUf = frequency of posting on social media.

**Supplementary Figure F2.** Percentage of variance explained through common additive genetic and environmental factors or phenotype-specific effects based on the common pathway model.


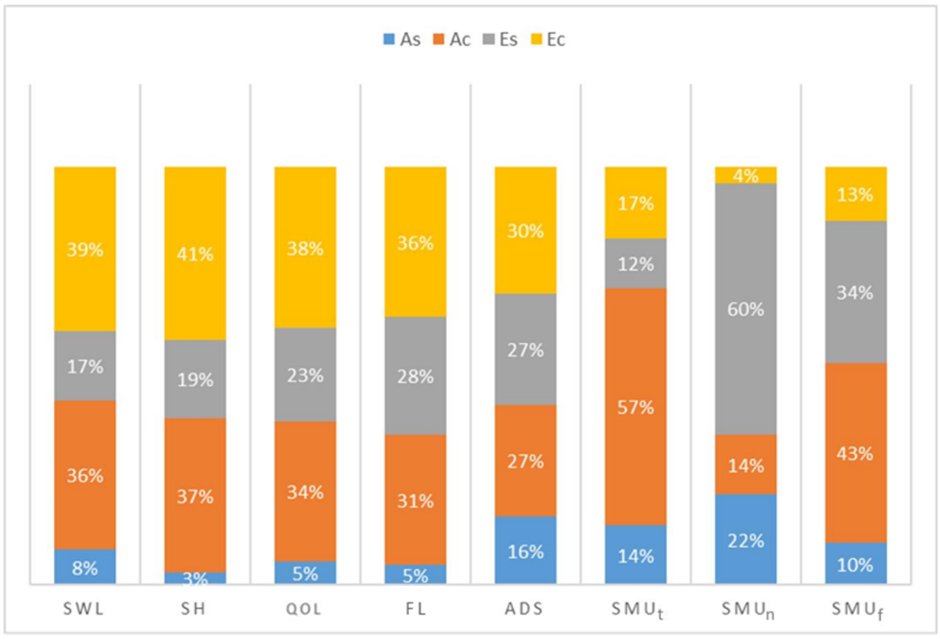


*Note.* Ac = percentage of total additive genetic variance explained by common genetic effects, As = percentage of total variance explained by additive genetic effects specific for a given phenotype, Ec = percentage of the total variance explained by common unique environmental effects, Es = percentage of total variance explained by unique environmental effects specific for a given phenotype. SWL = satisfaction with life, SH = subjective happiness, QoL = quality of life, FL = flourishing, ADS = anxious-depressive symptoms, SMUt = time spent (daily) on social media, SMUn = number of social media accounts owned, SMUf = posting on social media.

**Supplementary Figure F3.** Percentage of variances explained through common factor or phenotype-specific effects based on the common pathway model, recalculated with log10 transformed ADS and SMU_t_ scores, as a part of our post-hoc sensitivity analysis.

**
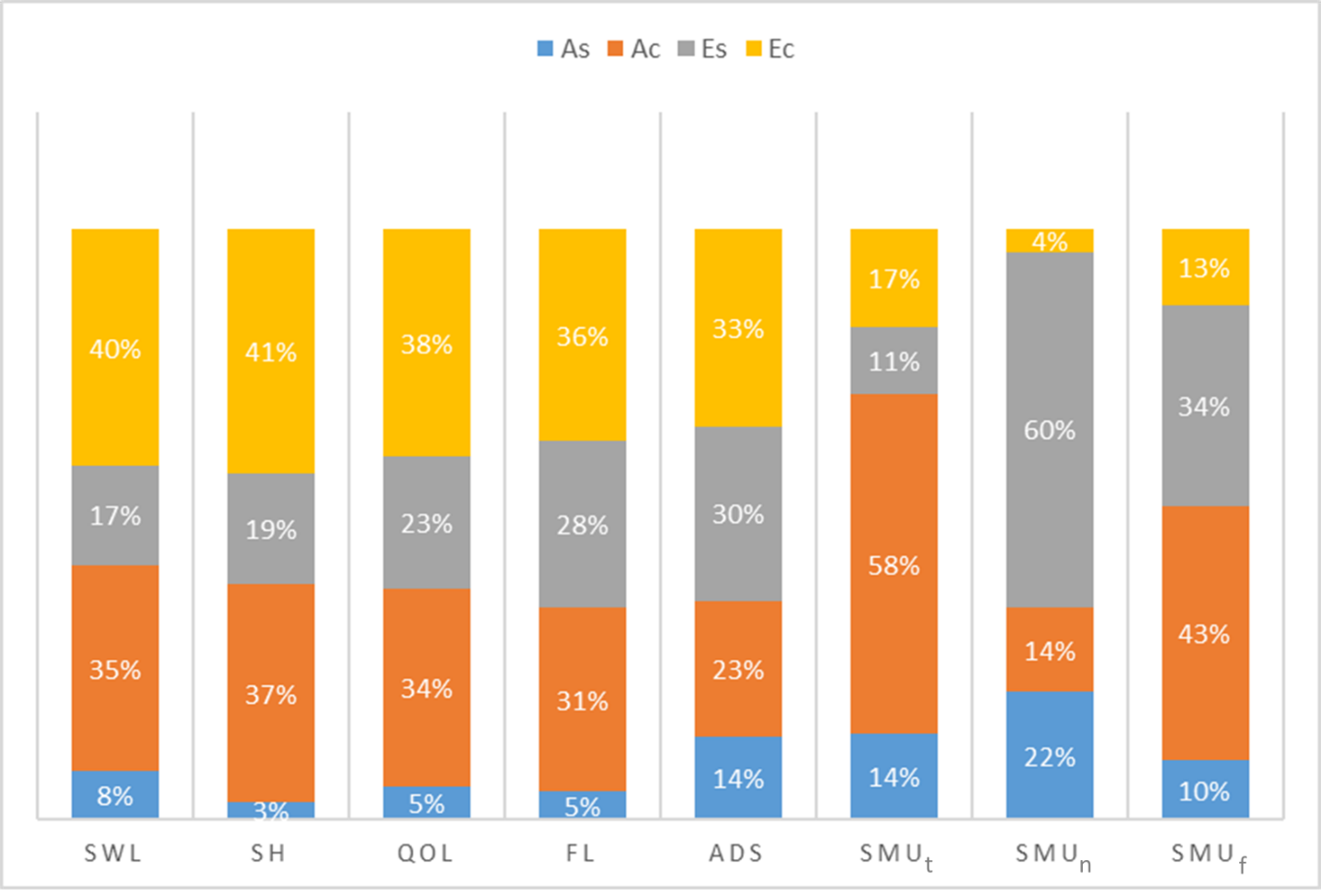
**

*Note.* Ac = percentage of the total additive genetic variance explained due to the common genetic effects for a given phenotype, As = percentage of total variance explained by the additive genetic effects specific for a given phenotype, Ec = percentage of the total variance explained by the common unique environmental effects for a given phenotype, Es = percentage of total variance explained by the unique environmental effects specific for a given phenotype. SWL = satisfaction with life, SH = subjective happiness, QoL = quality of life, FL = flourishing, ADS = anxious-depressive symptoms, SMU_t_ = time spent (daily) on social media, SMU_n_ = number of social media accounts owned, SMU_f_ = posting on social media.

**Supplementary results from the common pathway model**

The common pathway model with AE variance components provided a better fit than the model with all ACE components based on the chi-square differences test (*p*-value > 0.01) and the AIC values between the two models (174376.46 versus 174387.27, respectively). The Cholesky AE model provided a better fit than the common pathway AE model (173955.43 versus 174376.46, respectively). The common pathway model was used to provide a more fine-grained decomposition of the estimated variance components (i.e., through common and specific effects for each phenotype). The best-fitting common pathway model is shown in Supplementary Figure F1. The phenotypic correlation between the common WB and SMU factors was -0.05 (-0.08, -0.01). The standardized additive genetic (A) and unique environmental variance (E) for the common WB factor were 47% and 53%, respectively. For the common SMU factor, the standardized additive genetic (A) and the unique environmental variances (E) for the SMU factor were 77% and 23%, also in their respective order. The genetic and environmental influences on the covariance between the common WB and SMU factors were 70% and 30%, respectively. A non-significant genetic correlation of *r* = -0.06 (-0.13, 0.02) and a nonsignificant environmental correlation of *r* = -0.04 (-0.14, 0.06) were found between common WB and SMU factors as well. The total variance in the WB and ADS phenotypes was mostly due to common additive genetic effects (between 27% and 37% of the total variance) and not due to 18 phenotype-specific effects (between 3% and 16% of the total variance; see Supplementary Figure F2). For the non-shared environmental effects, the common effects (between 30% and 41% of the total variance) also played a more central role and not the phenotype-specific effects (between 17% to 28%).

SMU_t_ was predominantly determined by common additive genetic effects (57% of the total variance), and not as much through the phenotype-specific effects (only 14% of the total variance). The common and phenotype-specific non-shared environmental effects were similar, and they explained a smaller portion of the total variance in SMU_t_ (17% and 12% of the total variance, respectively). Variance in SMUf was largely determined by common (43%) and less by specific additive genetic factors (10%). The common non-shared environmental effects were smaller than the phenotype-specific counterparts (13% and 43% of the total variance, respectively). Different from the other two SMU variables, the variance in SMU_n_ was largely determined by phenotype-specific environmental effects (60% of the total variance), while the remaining factors played a more limited role. These were common non-shared environmental, and common and specific additive genetic factors, explaining 4%, 14%, and 22% of the total variances, respectively.
